# Serological response and diagnostic value of recombinant *candida* cell wall protein enolase, phosphoglycerate kinase, and β-glucosidase

**DOI:** 10.3389/fmicb.2015.00920

**Published:** 2015-09-10

**Authors:** Zheng-xin He, Jing Chen, Wei Li, Yan Cheng, Hai-pu Zhang, Li-na Zhang, Tian-wen Hou

**Affiliations:** Department of Clinical Laboratory, Bethune International Peace Hospital of PLAShijiazhuang, China

**Keywords:** invasive candidiasis, serodiagnosis, serological response, enolase, phosphoglycerate kinase, β-glucosidase

## Abstract

There are no specific signs and symtoms for invasive candidiasis (IC), which makes its diagnosis a challenge. Efforts have been made for decades to establish serological assays for rapid diagnosis of IC, but none of them have found widespread clinical use. Using a systemic candiasis murine model, serological response to recombinant proteins of enolase (rEno1), phosphoglycerate kinase (rPgk1), and β-glucosidase (rBgl2) were evaluated and rEno1 was found to possess the strongest immunoreactivity, followed by rPgk1 and rBgl2. Likewise, IgG antibody titers to rEno1, rPgk1, and rBgl2 in the positive sera of proven IC patients were determined by ELISA. Results show anti-rEno1 antibody possesses the highest titer, followed by rPgk1 and rBgl2. Antibodies against rEno1, rPgk1, and rBgl2 were detected by ELISA tests in a group of 52 proven IC patients or 50 healthy subjects, The sensitivity, specificity, positive and negative predictive values were 88.5, 90.0, 90.2, and 88.2% for anti-rEno1 detection, 86.5, 92.0, 91.8, and 86.8% for anti-rPgk1 detection, and 80.8, 90.0, 89.4, and 81.8% for anti-rBgl2 detection, respectively. The data clearly demonstrate that the recombinant proteins of Eno1, Pgk1, and Bgl2 are promising candidates for IC serodiagnosis. There's great possibility that the recombinant Eno1 will be more applicable in serodiagnosis and vaccine research on account of its strong serological response.

## Introduction

Invasive candidiasis (IC) continues to be a life-threatening infectious disease affecting an ever-increasing number of hospitalized patients and, of particular concern, causing considerably high morbidity and mortality. In the USA, Candida species is among the five major pathogens leading to nosocomial blood stream infections (BSIs) and causing 8–10% of nosocomial BSIs (Pappas, 2006; Pfaller and Diekema, 2007; Morrissey, 2013; Sievert et al., 2013). Though a variety of Candida species can produce invasive infection, *Candida albicans* (*C. albicans*) continues to be identified as a leading pathogen. The risk of candidiasis is increased by some factors mainly including broad-spectrum antibacterial therapy, intense myelosuppression, and cytotoxic therapies, recent gastrointestinal surgery and the presence of central venous access devices (Pappas, 2006).

The identification of patients at risk of IC is complicated in that there are usually no specific clinical signs and symptoms. Recovery of Candida species from blood specimen is considered as the standard method for candidemia diagnosis. However, the low sensitivity (approximately 50%) and positive results may appear only in the advanced stage of infection which dramatically hampers its role in clinical practice (Yera et al., 2001). Though the germ tube test has been performed successfully on samples collected directly from positive blood cultures, rather than waiting for Candida colonies to grow on agar plates (Terlecka et al., 2007; Sheppard et al., 2008), the long duration is still an overwhelming disadvantage of blood culture.

Efforts have been made for decades to establish serological diagnosis assay to identify IC patients. Detection of antigens from the infecting agent in host's samples is considered as one of the practical strategies. These antigenic molecules mainly include (1,3)-D-glucan (BDG) (Ostrosky-Zeichner et al., 2005; Pickering et al., 2005; Cuetara et al., 2009), galactomannan (Sendid et al., 1999), enolase (Walsh et al., 1991), aspartyl proteinase (Morrison et al., 2003), and D-arabinitol (Christensson et al., 1999; Yeo et al., 2000). Serological tests detecting candida cell wall components, such as galactomannan and BDG, have become widely used diagnostic tools and commercially available. Antigen detection has the advantage of high specificity but often lack the desired level of sensitivity required for a definitive diagnosis. The main reason for this may be that most of the circulation pathogen antigens are usually cleared out from the blood circulation rapidly since the occurrence of infection. In addition, false positive results may occur in antigen-based detection test because of the contaminants in sample preparations or patients suffering from certain bacterial infections (Hope et al., 2005; Mennink-Kersten et al., 2008).

Consideration of patient's antibody response provides another option for IC diagnosis. Eno1, Pgk1, and Bgl2 are well-documented proteins located on the cell surface of *C. albicans*. These proteins possess high conservation during species evolution and their loose association with the cell wall might facilitate the production of antibodies by *C. albicans*-infected hosts (Montagnoli et al., 2004; Pitarch et al., 2004, 2006). Based on immunoproteomic approach using two-dimensional electrophoresis followed by quantitative Western blotting and mass spectrometry, Pitarch et al. (2006) demonstrated that Eno1, Pgk1, and Bgl2 were the independent predictors of systemic candidiasis (SC) and valuable candidates for future vaccine development. These enzymes show strong antibody response to the SC patients' sera, even in the immunocompromised hosts.

In the present study, we evaluate the immunoreactivity of recombinant *C. albicans* proteins of Eno1 (rEno1), Pgk1 (rPgk1), and Bgl2 (rBgl2) by a systemic candiasis murine model. We also report the serodiagnosis of infection by invasive Candida species using ELISA to detect specific antibodies against rEno1, rPgk1, and rBgl2. The objective of this study is to explore the potential use of rEno1, rPgk1, and rBgl2 in the diagnosis of IC to further expand our knowledge of IC control.

## Materials and methods

### Candida strain and growth conditions

*C. albicans* strain SC5314 was routinely grown in YPD medium (1% yeast extract, 2% peptone, 2%D-glucose) at 35°C, 5% CO_2_.

### Study population and serum specimens

All patients were admitted to the Bethune International Peace Hospital, Shijiazhuang, China, from October 2011 to December 2013. Following the criteria made by the European Organization for the Research and Treatment of Cancer/Mycoses Study Group [EORTC/MSG] (Ascioglu et al., 2002), the 102 subjects enrolled in this study were classified to two groups: proven IC patients group (*n* = 52) and control individuals (*n* = 50). For proven IC subjects, the serum samples were obtained retrospectively within 24 h after positive culture results, which assured uniformity and that all subjects had active disease at the time of enrollment. To provide data on assay specificity, the control individuals had similar age and sex distribution to the proven IC patients. Serum was separated, aliquoted, and stored at 2 ~ 8°C for up to 48 h or frozen at −80°C until tested.

The study protocol was approved by the Ethics Committee of Bethune International Peace Hospital and informed consent was obtained from all patients included in the study. All sera were analyzed in a blinded fashion. Data including age, primary condition, and clinical stage were obtained from the clinical records. Base-line characteristics of the proven IC patients are shown in Table 1.

**Table 1:** **Base-line characteristics of the 52 patients with invasive candidiasis included in the study**.

**Patient No**.	**Sex^a^**	**Age**	**Hospital ward**	**Underlying condition**	**Site of *Candida* isolation^b^**	**Isolated *Candida***
1	F	77	Respiratory	Pneumonia	Blood	*C. albicans*
2	M	93	Respiratory	Pneumonia; Cardiac carcinoma	Blood	*C. albicans*
3	F	33	Surgery	Acute pancreatitis	Blood	*C. tropicalis*
4	M	72	ICU	Gastric carcinoma; Acute peritonitis	Blood	*C. glabrata*
5	M	80	Respiratory	Pneumonia	Blood	*C. tropicalis/*
						*C. parapsilosis*
6	M	66	ICU	Intestinal obstruction	Blood	*C. albicans*
7	M	79	Cardiology	Coronary heart disease	Blood	*C. glabrata*
8	M	81	ICU	Cardiac carcinoma	Blood	*C. tropicalis*
9	M	87	Respiratory	Chronic bronchitis	Blood	*C. lusitaniae*
10	M	72	Hematology	Chronic myelocytic leukemia	Blood	*C. albicans*
11	M	84	Endocrinology	Diabetes	Blood	*C. krusei*
12	F	34	ICU	Septic shock	Blood	*C. albicans*
13	M	31	ICU	Septic shock	Blood	*C. albicans*
14	M	70	Respiratory	Respiratory failure	Blood	*C. lusitaniae*
15	M	88	Respiratory	Respiratory failure	Blood	*C. krusei*
16	M	61	ICU	Adenocarcinoma	Blood	*C. parapsilosis*
17	M	31	Burns unit	Burns	Blood	*C. albicans*
18	F	85	Surgery	Colorectal carcinoma	Blood	*C. albicans*
19	F	81	ICU		Blood	*C. glabrata*
20	F	64	Infectious disease	Hepatic Cirrhosis	Blood	*C. glabrata*
21	F	52	ICU	Asthma	Blood	*C. albicans*
22	M	72	Respiratory	Pneumonia	Blood	*C. krusei*
23	M	26	ICU	Septic shock	Blood	*C. albicans*
24	M	28	Burns unit	Burns	Blood	*C. albicans*
25	M	81	ICU	Cardiac carcinoma	Blood	*C. tropicalis*
26	M	73	ICU	Gastric carcinoma	Blood	*C. tropicalis*
27	M	52	Burns unit	Burns	Blood	*C. tropicalis*
28	F	49	ICU	Multiple organ failure	Blood	*C. tropicalis*
29	M	58	Surgery	Surgery	Blood	*C. glabrata*
30	M	62	Surgery	Small intestine fistula	Blood	*C. parapsilosis*
31	M	73	Respiratory	Pneumonia	Blood	*C. albicans*
32	M	64	ICU	Gastric carcinoma	Blood	*C. tropicalis*
33	F	41	Hematology	Leukemia	Blood	*C. albicans*
34	F	29	Respiratory	Respiratory failure	Blood	*C. parapsilosis*
35	F	58	Surgery	Cardiac carcinoma	Ascites	*C. albicans*
36	M	32	Surgery	Acute pancreatitis	Ascites	*C. parapsilosis*
37	M	38	Surgery	Peritonitis	Ascites	*C. albicans*
38	F	61	Surgery	Colorectal carcinoma	Ascites	*C. parapsilosis*
39	M	31	ICU	Abdomen trauma	Ascites	*C. tropicalis*
40	M	21	ICU	Abdominal stab wound	Ascites	*C. parapsilosis*
41	M	75	ICU	Peritonitis	Ascites	*C. albicans*
42	F	61	Respiratory	Cardiac carcinoma	Ascites	*C. glabrata*
43	F	59	Respiratory	Abdominal stab wound	Ascites	*C. parapsilosis*
44	M	69	Surgery	Peritonitis	Ascites	*C. glabrata*
45	F	32	Surgery	Peritonitis	Ascites	*C. albicans*
46	M	82	Surgery	Small intestine carcinoma	PL	*C. parapsilosis*
47	F	63	Surgery	Esophageal cancer	PL	*C. albicans*
48	M	88	Digestive disease	Gastric carcinoma	PL	*C. albicans*
49	F	52	Surgery	Right shoulder arthritis	JF	*C. guilliermondii*
50	F	82	Respiratory	Pneumonia	JF	*C. albicans*
51	F	60	Surgery	Craniocerebral trauma	CSF	*C. albicans*
52	M	85	Surgery	Subarachnoid hemorrhage	CSF	*C. albicans*

a *M, male; F, female*.

b *PL, pleural liquid; JF, joint fluid; BALF, bronchoalveolar lavage fluid; CSF, cerebrospinal fluid*.

### Generation of recombinant Eno1, Pgk1, and Bgl2

As is shown in Table 2, primers were designed to clone and express full length protein with the software of Primer Premier 5.0. PCR products were cloned into the pET-30a (+) expression vector (Merckmillipore, Germany). All inserts were confirmed by DNA sequencing. The plasmids were transformed into *Escherichia coli* BL21 (DE3) competent cells (Transgen Biotech, Beijing, China). Expression of recombinant antigens was induced by isopropyl-β-D-thiogalactopyranoside (IPTG). His6-tagged recombinant proteins were confirmed by 12% SDS-PAGE. Recombinant antigens were purified from cell-free supernatants by chromatography on Ni^2+^ nitrilotriacetic acid-agarose (His-trap HP) in accordance with the manufacturer's instructions (GE Healthcare, USA).

**Table 2:** **Oligonucleotide primer sequences used in this study**.

**Primer^a^**	**Genebank accession No**.	**Sequence^b^ (5^′^ - 3^′^)**	**Restriction enzyme**
Eno1_F	NW_139596	CCGGATCCATGTCTTACGCCACTAAAATC	BamH I
Eno1_R		GCCTCGAGTTACAATTGAGAAGCCTTTTG	Xhol I
Pgk1_F	NW_139621	GCGGATCCTCATTATCTAACAAATT	BamH I
Pgk1_R		TACTCGAGGTTTTTGTTGGAAAGA	Xhol I
Bgl2_F	NW_139425	AAGGATCCATGCAAATCAAATTCTTGAC	BamH I
Bgl2_R		CGCTCGAGTTAGTTGAATTTACAGTCAA	Xhol I

a Eno, enolase; Pgk, phosphoglycerate kinase; Bgl, β-glucosidase; F, forward; R, reverse;

b *Underlined, restriction sites*.

Triton X-114 liquid phase separation was carried out to remove the contaminated endotoxin (LPS) in the recombinant proteins. The recombinant protein solutions (1 mL) were mixed with 100 μL 10% (w/v) Triton X-114 (sigma) by vigorous vortexing. The mixtures were placed in an ice bath for 10 min to ensure a homologous solution. After vortexing, the samples were incubated at 37°C for 10 min to form the two phases. Then, the samples were centrifuged for 1 min with a microcentrifuge at 25°C in an incubator. Fractions containing purified recombinant proteins were pooled, dialyzed against PBS and stored at −80°C.

### Murine model of systemic candidiasis

Specific pathogen free (SPF) BALB/c female mice weighing from 18 ~ 20 g (Vital River, China) were chosen to establish the murine model of SC. Animal welfare and experimental procedures were approved by the Bethune International Peace Hospital Animal Care and Use Committee. Protocols were conducted in strict accordance with the Guide for the Care and Use of Laboratory Animals (National Research Council (US) Institute for Laboratory Animal Research, 1996). Efforts were made to minimize the animals used and their suffering. Before the pathogen challenge, mice were injected intraperitoneally (i.p.) with Cyclophosphamide (Jiangsu Hengrui Medicine Co., Ltd., China) at a dose of 100 mg/kg. Four days later, mice were injected i.p. with a dose of 1 × 10^6^ CFU of *C. albicans* SC5314 (in 0.1 mL) or an equivalent amount of sterile saline as control. Three randomly chosen mice from each group were humanely sacrificed on the day of 0, 1, 2, 4, 6, 8, 12, 16, 20, 24, and 30 after pathogen administration. Blood was collected by cardiac puncture and was allowed to clot on ice. The serum was separated from the clot by centrifugation and stored at −80°C. Pathogen loads in kidney, lung, liver, and spleen of infected mice were determined (homogenized in saline) at different time points during the course of infection. Kidneys were dissected and processed to histological analysis (formalin fixed).

### Preparation of *C. albicans* protein extract

Yeast cells were harvested and washed with pre-cold sterile saline and then incubated at 30°C in a solution containing 30 g/ml Glusulase (sigma), 1 M sorbitol and 1% 2-mercaptoethanol (up to 5 × 10^8^ cells/ml) until over 90% protoplasts were obtained. After a vigorous vortexing and adding 1 mM PMSF, an ultrasonication process was conducted to disrupt the protoplasts. The mixture was centrifuged at 4°C and the supernatant was sterile filtered (0.2-mm pore) to remove residual debris and intact cells.

### ELISA assay

Preliminary checkerboard titration experiments were performed to determine the optimal concentration of antigen by comparing the known positive and negative human sera.

For measuring the serum antibody titers of SC mice, rEno1, rPgk1, rBgl2 were used to coat 96-well microtiter plates (JET BIOFIL, China). Protein extract of *C. albicans* was used as positive control, and protein extract of *E. coli* and BSA were used as negative controls. The concentrations of these proteins were adjusted to 500 ng/mL before use. Unbound antigens were removed by washing with PBS-T (PBS containing 0.05% Tween-20). The wells were blocked with 3% BSA/PBS-T over night at 4°C. Serial two-fold diluted sera of mice were used as the test antibody, and peroxidise conjugate goat anti-mouse antibody (1:10,000 diluted) was used as the secondary antibody. Titers were defined as the highest serum dilution with a positive result.

For probing with human serum, wells of microtiter plates were coated, blocked and washed as described above. Pre-diluted human sera (1:400 for anti-rEno1 probing, 1:200 for anti-rPgk1 probing and 1:100 for anti-rBgl2 probing) was added to the plates and 1:10,000 diluted peroxidise conjugate goat anti-human antibody was used as the secondary antibody. The antibody titers of proven IC patients were determined by using serial 10-fold diluted sera as the test antibody.

After incubation and washing, 3′, 3′, 5′, 5′-Tetramethylbenzidine (TMB) was added into the wells as the substrate solution. After 10 min of incubation at 37°C, the reaction was terminated with 2 N H_2_SO_4_, and the microplates were read at a wavelength of 450 nm using an ELISA reader (VersaMax plate reader, Molecular Devices Co.).

### Western blot

The recombinant proteins of rEno1, rPgk1, and rBgl2 were transferred to PVDF membrane (merckmillipore, Germany) after 12% SDS-PAGE electrophoresis. Membrane was blocked with Tris buffer (pH 7.5) containing 3% BSA. Next, a 1:400 diluted mice sera mixture, which was 1:1 blended from the collection on the 20th and 24th day after *C. albicans* administration, was incubated with the membrane at room temperature for 1 h. Then, the membrane was incubated with peroxidase-conjugated goat anti-mouse IgG (1/10000). Western blot was developed using dianilinobenzene (DAB) substrate (Solarbio, China).

### Statistical analysis

All data are given as mean plus or minus SD. Statistical significance of differences was assessed with Student's *t*-tests, with a *P* < 0.05 considered to indicate statistically significant differences. Sensitivity of diagnostic techniques was calculated from proven IC cases. Specificity was calculated from control group. Statistical analysis was performed using GraphPad Prism 5.0.

## Results

### Generation of recombinant protein

**Figure 1:**
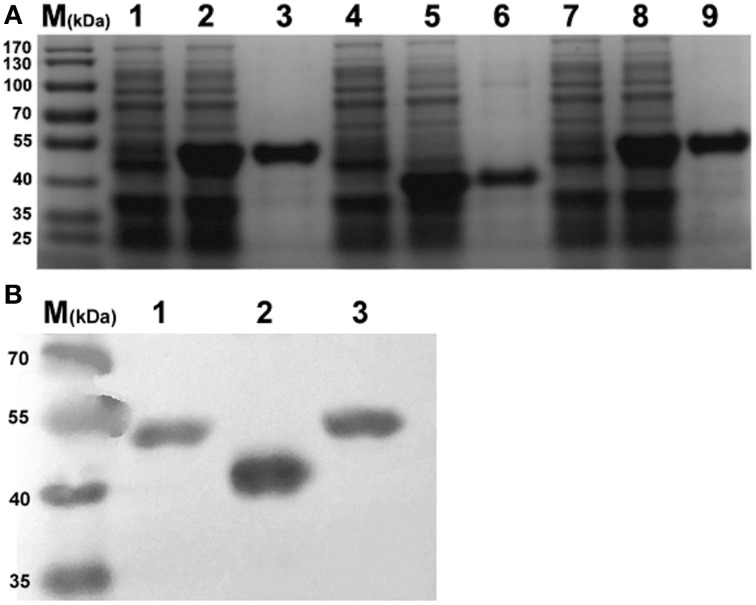
**SDS-PAGE and Western-blot identification of recombinant Eno1, Bgl2, and Pgk1. (A)** SDS-PAGE identification. Lanes: 1, pET30a-Pgk1 in *E. coli* BL21; 2, pET30a-Pgk1 in *E. coli* BL21, induced by IPTG; 3, purified recombinant Pgk1(46 kDa); 4, pET30a-Bgl2 in *E. coli* BL21; 5, pET30a-Bgl2 in *E. coli* BL21, induced by IPTG; 6, purified recombinant Bgl2(33–37 kDa); 7, pET30a-Eno1 in *E. coli* BL21; 8, pET30a-Eno1 in *E. coli* BL21, induced by IPTG; 9, purified recombinant Eno1(47 kDa). Molecular markers (in kDa) of standard proteins are to the left. **(B)** Identity the recombinant Pgk1(lane 1), Bgl2(lane 2), and Eno1(lane 3) by Western blotting using peroxidise -conjugated 6 × His monoclonal antibody, Molecular markers (in kDa) of standard proteins are to the left.

For purification of rEno1, rPgk1, and rBgl2, *E. coli* cells were lysed, and the extracts were subjected to centrifugation to obtain soluble supernatants and pellet fractions. The recombinant proteins were mainly contained in the soluble fraction and purified from this fraction by affinity chromatography using an agarose/Ni-nitrilotriacetic acid column as described in Section Materials and Methods. As expected, the eluted proteins show single bands at the expected molecular mass (Eno1 = 48 kDa, Pgk1 = 46 kDa, and Bgl2 = 33–37 kDa) (Pitarch et al., 2006) on SDS/PAGE (Figure 1A). The identity of the proteins was further established by Western blotting using HRP-labeled 6 × His monoclonal antibody (Figure 1B). The recombinant proteins were made LPS-free (< 5 EU/mL) by the method of Triton X-114 liquid phase separation.

### Establishment of the murine model of systemic candidiasis

**Figure 2:**
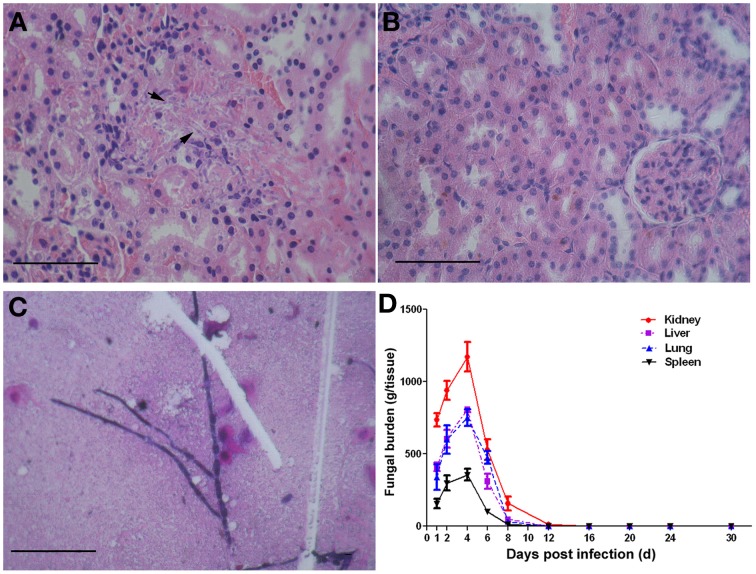
**The murine model of systemic candidiasis**. Mice were injected i.p. with a dose of 1 × 10^6^ CFU of *C. albicans* SC5314 or sterile saline as control. On the day of 0, 1, 2, 4, 6, 8, 12, 16, 20, 24, and 30 post infections, three mice of each group were randomly chosen and sampled. Disease progression was measured in terms of vital organ burdens and kidney histopathology. Fungal cells (arrowheads) are obvious in the kidneys of mice infected with *C. albicans* SC5314 **(A)**, and control kidneys showed no evidence of fungal cells or immune cell infiltrates **(B)**. Fungal hyphae can be observed in peritoneal fluid of infected mice **(C)**. Scale bar represents 100 mm. The vital organ fungal burdens reached their peak on the 4th day and ceased to exist on the 12th day post infection **(D)**.

Within 6 h after *C. albicans* SC5314 administration, the mice show typical symptoms of infection including sweat, physical inactivity compared with the control mice. These infectious symptoms fade away about 4 days later. Since the kidneys are reported as the most heavily colonized organs in the mouse intravenous challenge model (Spellberg et al., 2005; MacCallum, 2009), we chose this organ as an indicator of the mouse intraperitoneal challenge model for successful infection in the present study. The kidneys of *C. albicans*-infected mice contained filamentous fungal cells which were associated with leukocyte infiltrates (Figure 2A), but there was no evidence of fungal cells or immune cell infiltrates (Figure 2B) in the kidneys from the control mice. Peritoneal fluid was used for smear and the growth of fungal hyphae could be easily found under a microscope (Figure 2C). The fungal densities in kidney, lung, liver, and spleen of infected mice at different time points are presented in Figure 2D. The number of fungal colonies gradually increases from the 1st day after *Candida* administration. On the 4th day, the fungal loads in the vital organs reached their peaks and then gradually decreased. On the 12th day, the fungi ceased to exist in the lung, liver, and spleen, and the fungal load in kidney was dropped to 11 ± 5 CFU/g tissues.

### Serological response to rEno1, rPgk1, and rBgl2

**Figure 3:**
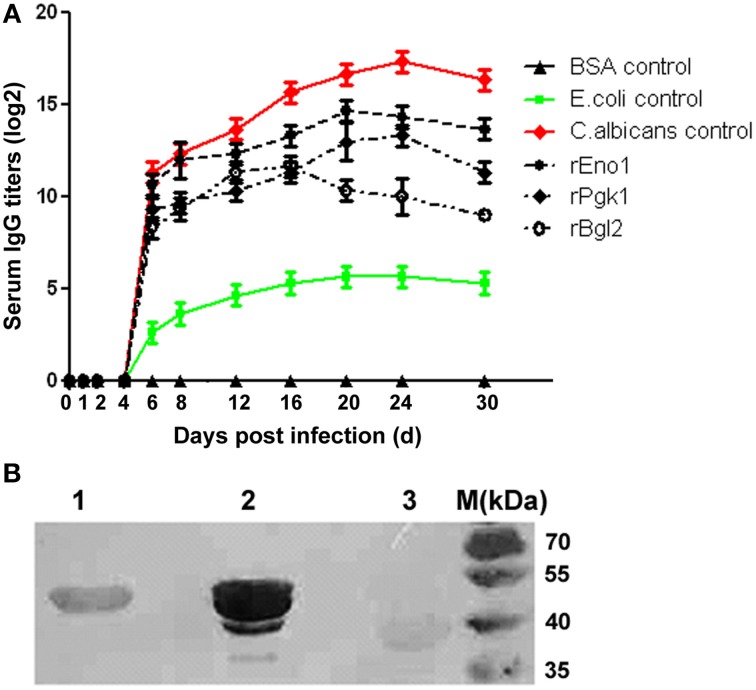
**Serological response of *C. albicans* infected mice to recombinant protein Eno1, Pgk1, and Bgl2**. **(A)** The kinetics of IgG antibody titers to rEno1, rPgk1, and rBgl2 were measured by ELISA, mice sera were 1:100 pre-diluted before detection. Protein extract of *C. albicans* was used as positive control, and protein extract of *E. coli* and BSA were used as negative controls. **(B)** Using the mixed mice sera collected from the 20th and 24th day post infection as the first antibody (1:400 pre-diluted), Western-blot show rEno1 possess the strongest immunoreactivity, followed by rPgk1 and rBgl2.

To investigate the serological response of *C. albicans* infected mice to rEno1, rPgk1, and rBgl2, the kinetic changes of IgG antibody titers to the recombinant proteins were measured by ELISA assay. Experimental results show that the mice with SC can effectively generate IgG antibodies against rEno1, rPgk1, and rBgl2 (Figure 3A). On the 6th day post infection, antibodies against rEno1, rPgk1, and rBgl2 can be detected in the mice sera and the antibody titers increasing gradually over time. Antibody titers to rEno1, rPgk1, and rBgl2 reached their peak on the day of 20, 24, and 16 post infection and then slightly dropped down. The highest titer is about 1:32,768 (1:2^15^) for rEno1, 1:8192 (1:2^13^) for rPgk1 and 1:4096 (1:2^12^) for rBgl2.

Using the mixed mice sera collected from the 20th and 24th day post infection as the first antibody, immunoblotting analysis shows similar results compared with those from the ELISA tests. The recombinant protein rEno1 was found to possess the strongest immuno-reactivity, followed by rPgk1 and rBgl2 (Figure 3B).

### Detection of IgG antibodies in human sera

**Figure 4:**
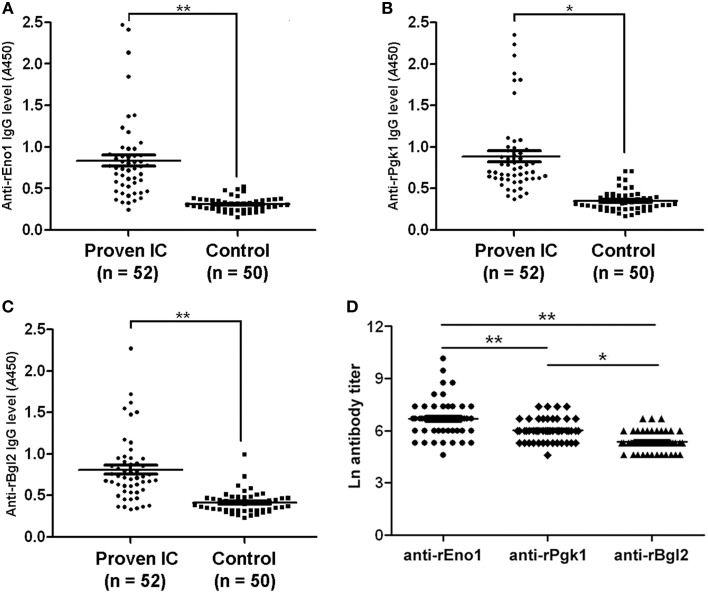
**Antibody levels and titers in study patients**. The levels of anti-rEno1 **(A)**, anti-rPgk1 **(B)**, and anti-rBgl2 **(C)** in human sera were determined by ELISA and human sera were pre-diluted before detection (1:400 for rEno1, 1:200 for rPgk1, and 1:100 for rBgl2). The levels of anti-rEno1, anti-rPgk1 and anti-rBgl2 in proven IC patients were higher than control group. **(D)** Ln antibody titers in proven IC patients. Anti-rEno1 antibody possesses the highest titer, followed by rPgk1 and rBgl2. Each symbol represents antibody level (or titer in **D**) of individual human serum. The horizontal lines indicate the mean ± SEM for each group. Means were compared by Students *t*-test: ^**^P < 0.001, ^*^P = 0.001.

Figure 4 shows the results obtained with the serum specimens drawn from the 52 patients and 50 control subjects who were tested by ELISA for the detection of IgG antibodies. The median of anti-rEno1 antibody absorbance for sera from proven IC patients (median 0.755; interquartile range, 0.527–0.897) was significantly higher than that from control individuals (median 0.306; interquartile range, 0.254–0.355; *P* < 0.001) (Figure 4A). Likewise, proven IC patients had a greater prevalence of seropositive anti-rPgk1 antibody (median 0.715; interquartile range, 0.613–0.954) than control individuals (median 0.313; interquartile range, 0.256–0.392; *P* = 0.001) (Figure 4B). For rBgl2, there is also a higher antibody absorbance in proven IC patients (median 0.721; interquartile range, 0.557–0.893) than in control individuals(median 0.398; interquartile range, 0.320–0.468; *P* < 0.001) (Figure 4C). The cut-off values for serum anti-rEno1, anti-rPgk1, and anti-rBgl2 were set by receiver operating characteristic (ROC) curves with *A* = 0.436 for anti-rEno1, *A* = 0.537 for anti-rPgk1, and *A* = 0.529 for anti-rBgl2. A patient serum was regarded as positive when ELISA result was above the cut-off point. As is shown in Table 3, there is the sensitivity, specificity, and positive and negative predictive values (PPV and NPV) calculated per patient for the anti-rEno1, anti-rPgk1, and anti-rBgl2 antibody detection tests. When the cutoff values mentioned above were used, the sensitivity, specificity, PPV, and NPV were 88.5, 90.0, 90.2, and 88.2% for anti-rEno1 detection, 86.5, 92.0, 91.8, and 86.8% for anti-rPgk1 detection, and 80.8, 90.0, 89.4, and 81.8% for anti-rBgl2 detection, respectively. Serological and microbiological surveillance of proven IC patients are reported in Table 4.

**Table 3:** **Sensitivity, specificity, and predictive values for the detection of anti-rEno1, anti-rPgk1 and anti-rBgl2^a^**.

	**Cutoff value**	**Sensitivity, %**	**specificity, %**	**PPV^b^, %**	**NPV^c^, %**
Anti-rEno1	0.436	88.5	90.0	90.2	88.2
Anti-rPgk1	0.537	86.5	92.0	91.8	86.8
Anti-rBgl2	0.529	80.8	90.0	89.4	81.8

a Results are calculated per patient according to the results of an analysis of 52 serum samples from 52 proven IC patients and 50 serum samples from 50 controls;

b PPV, positive predictive value;

c *NPV, negative predictive value*.

**Table 4:** **IgG antibodies and microbiological surveillance of proven IC patients**.

**Pathogen**	***n***	**No. (%) positive**		
		**Anti-rEno1**	**Anti-rPgk1**	**Anti-rBgl2**
*C. albicans*	22	20 (90.1)	20 (90.1)	21 (95.5)
*C. tropicalis*^a^	9	8 (88.9)	7 (77.8)	6 (66.7)
*C. parapsilosis*^a^	9	8 (88.9)	7 (77.8)	8 (88.9)
*C. glabrata*	7	6 (85.7)	7 (100)	5 (71.4)
*C. lusitaniae*	2	2 (100)	1 (50.0)	1 (50.0)
*C. krusei*	3	2 (66.7)	3 (100)	1(33.3)
*C. guilliermondii*	1	1 (100)	1 (100)	1 (100)
Total	53	47 (88.7)	46 (86.8)	43 (81.1)

a *one patient was confirmed to be infected with two Candida species of C. tropicalis and C. parapsilosis*.

In this study, IgG antibody titers to rEno1, rPgk1, and rBgl2 in the positive sera of proven IC patients were determined by ELISA. As was observed in the systematic infection murine model, anti-rEno1 antibody possesses the significant higher Ln titer than anti-rPgk1 (6.90 ± 0.98 vs. 6.12 ± 0.58; *P* < 0.001) or anti-rBgl2 (6.90 ± 0.98 vs. 5.53 ± 0.50; *P* < 0.001), and anti-rPgk1 antibody Ln titer is significantly higher (*P* = 0.001) than anti-rBgl2 (Figure 4D).

## Discussion

There is an increasing interest in the development of new, reliable, and simple diagnostic tests for the diagnosis of IC. It has been demonstrated that detection of antibodies against cell wall antigens has diagnostic potency for IC (Clancy et al., 2008; Li et al., 2013).

In the present study, we generated recombinant proteins of three cell wall proteins, Eno1, Pkg1, and Bgl2, from *C. albicans* SC5314. By using rEno1, rPgk1, and rBgl2 as the antigens, we developed an indirect ELISA assay to detect the IgG antibodies against them and ELISA specificity was confirmed by block assay. Results revealed that recombinant antigens of Eno1, Pgk1, and Bgl2 from *C. albicans* reacted to sera from proven IC patients infected with *C. albicans* and other Candida species including *C. tropicalis, C. parapsilosis, C. glabrata, C. lusitaniae, C. krusei*, and *C. guilliermondii*. Several studies have achieved promising results in the diagnostic utility of the detection of antibodies against rEno1 (Laín et al., 2007; Clancy et al., 2008; Pitarch et al., 2008; Li et al., 2013). In 2006, Bgl2 and Pgk1 were described as novel antigens for SC dianosis (Pitarch et al., 2006). According to a report from Clancy et al. (2008) about antibody responses against 15 recombinant antigens from 12 proteins, IgG titers were detectable for anti-rEno1 in 98.3% (59/60) of participants with system candidiasis, for anti-rBgl2 in 95.0% (57/60), and for anti-rPgk1 in 89.8 ~ 91.5% (53 ~ 54/59). In this study, we got similar results (Table 3) and the sensitivity, specificity, positive, and negative predictive values were 88.5, 90.0, 90.2, and 88.2% for anti-rEno1 detection, 86.5, 92.0, 91.8, and 86.8% for anti-rPgk1 detection and 80.8, 90.0, 89.4, and 81.8% for anti-rBgl2 detection, respectively. Though obtaining multiple samples might increase the sensitivity, specificity, PPV, and NPV (Odabasi et al., 2004), we got remarkable results here based on single sample protocol according to which the serum samples were obtained retrospectively within 24 h after positive culture results. This indicates that the factors affecting the clinical performance of serological diagnostic tests are not only how to obtain the samples, but also when. Use of combinations of the ELISAs were recommended by some researchers, but the data (not shown) we got didn't show increased ability to detect infection in participants with IC when two or three ELISAs were combined, which might be attributed to the fact that the performance of each assay was remarkable enough.

IgG antibodies against rEno1, rPgk1, and rBgl2 can be detected in the serum of SC mice. The kinetics of antibodies against each recombinant antigen in the same model is different, which can be directly observed by immunoblotting and validated by titer determination of positive sera from proven IC patients. These results indicate that recombinant Eno1, Bgl2, and Pgk1 are different at the immunoreactivity with sera from SC mice or IC patients, even though they are effective immunogenes. Reasons for this complication may be: (1) Recombinant proteins cannot be fully equal to the natural ones and potential epitopes could be lost due to miss folding or a lack of correct post-translational modification, both of which may affect the conformational structure of the native protein. In support of this, Mochon et al. (2010) reported antigenicity of *in vitro* expressed Bgl2 without any glycosylation was likely to be different from the Bgl2 produced by *C. albicans* used in the 2D-PAGE immunoblots. (2) The immunogenicity for Eno1, Pgk1, and Bgl2 might be different in the Candida infected hosts; (3) Host serological response to pathogen antigen is limited by the range in protein abundance, and the abundances of Eno1, Pgk1, and Bgl2 are different in the cell wall of *C. albicans*. Enolase is one of the most abundant proteins in *C. albicans* (Sundstrom and Aliaga, 1992) as it accounts for about 0.7% of the total protein content of yeast and about 2% of hypha (Sundstrom et al., 1994). Better knowledge of serological response to different recombinant antigens is useful to develop a peptide or DNA vaccine against *C. albicans* and to identify selective serological diagnostic reagents. In this study, high serological response of recombinant Eno1 makes it possible to develop more sensitive and quantitative IgG detection methods in clinical practice for IC serodiagnosis. According to the murine model experimental data, high antibody levels remain after organ fungal burdens become undetectable. This might mean that patients would be tested as positive after successful anti-fungal treatment and we will further investigate how the serological profile changes during the treatment in the future.

Establishment of suitable animal model is one of the critical means of pathogenic biological research. To generate a murine model of SC, the researchers mostly employ intravenous inoculation (Louie et al., 1998; MacCallum, 2009). In this study, we injected *C. albicans* SC5314 into the peritoneal cavity of cyclophosphamide preconditioned immunocompromised mice to cause systemic infection. For a BALB/c mouse with 18 ~ 20 g body weight, the challenging dose is about 1 × 10^6^ CFU. A dose of 1 × 10^7^ CFU/mouse for intraperitoneal inoculation would kill the mice in 3 days according to our experimental data (not shown). Compared with the intravenous injection model, the intraperitoneal injection model has the advantage of easy operation. Besides successful systemic infection and immune challenge, a low dose of *C. albicans* challenge model (1 × 10^6^ CFU/mouse, i.p.) has no fatal damage to the mice and is suitable for serological studies that need relatively long-term observation.

### Conflict of interest statement

The authors declare that the research was conducted in the absence of any commercial or financial relationships that could be construed as a potential conflict of interest.
